# Reversed Scototaxis during Withdrawal after Daily-Moderate, but Not Weekly-Binge, Administration of Ethanol in Zebrafish

**DOI:** 10.1371/journal.pone.0063319

**Published:** 2013-05-13

**Authors:** Adam Holcombe, Adam Howorko, Russell A. Powell, Melike Schalomon, Trevor J. Hamilton

**Affiliations:** Department of Psychology, Grant MacEwan University, Edmonton, Alberta, Canada; Tulane University Medical School, United States of America

## Abstract

Alcohol abuse can lead to severe psychological and physiological damage. Little is known, however, about the relative impact of a small, daily dose of alcohol (*daily-moderate schedule*) versus a large, once per week dose (*weekly-binge schedule*). In this study, we examined the effect of each of these schedules on behavioural measures of anxiety in zebrafish (*Danio rerio*). Adult wild-type zebrafish were administered either 0.2% ethanol on a daily-moderate schedule or 1.4% ethanol on a weekly-binge schedule for a period of 21 days, and then tested for scototaxis (preference for darkness) during withdrawal. Compared to a control group with no alcohol exposure, the daily-moderate group spent significantly more time on the light side of the arena (indicative of decreased anxiety) on day two of withdrawal, but not day 9 of withdrawal. The weekly-binge group was not significantly different from the control group on either day of withdrawal and showed no preference for either the light or dark zones. Our results indicate that even a small dose of alcohol on a daily basis can cause significant, though reversible, changes in behaviour.

## Introduction

While moderate alcohol consumption is increasingly being touted as having significant health benefits [1], overconsumption of alcohol remains a worldwide problem that results in 2.5 million deaths each year [2]. Withdrawal from repeated alcohol exposure is debilitating and can cause increases in alcohol-seeking behaviours, anxiety, and seizure activity. The severity of withdrawal depends on prior intake levels and pattern of over-consumption. Two common patterns of over-consumption are chronic drinking and binge drinking. Chronic drinking involves frequent consumption of relatively low doses, and can lead to memory disorders, decreased immune system functioning, and cardiovascular, pancreatic, and digestive problems [3]. It is also associated with a decrease in psychological well-being [4], as well as neuroinflammation and neurodegeneration [5]. In animal research a chronic drinking schedule usually refers to constant ethanol exposure for long periods of time, or exposure for the majority of the day. Binge drinking (also termed “chronic intermittent ethanol (CIE) exposure” [6] in animal research) is a distinct form of over-consumption that involves infrequent consumption of relatively large doses of alcohol. Binge drinking is most common in humans aged 18–34 years, and can result in significant neurological and psychological changes, including cognitive impairment [3] and decreased overall quality of life [7]. Recent research with rodents has demonstrated that binge drinking impairs the extinction of fear responses via morphological changes in pyramidal neurons of the medial prefrontal cortex [8] and impairs prefrontal synaptic plasticity [9]. Both chronic and binge drinking have been studied in rodents [9], [10] and humans [11], with a few studies comparing the detrimental effects of each schedule. For example, chronic drinking in rats produces more damage to the liver than either acute or binge drinking [12]. However, the differential effects of moderate drinking versus binge drinking on withdrawal-induced behaviour in animals have not yet been investigated.

The zebrafish (*Danio rerio*) has become a popular model organism for behavioural and genetic studies [13], [14], and has proven to be a useful model for investigating human disease [15], [16]. Zebrafish larvae are especially amenable to genetic, pathological, and pharmacological studies because of their simple genome [17], [18], [19], [20]. Adult zebrafish have been employed to investigate anxiety, using basic behavioural paradigms such as the light-dark test for scototaxis (preference for dark areas; [21]) and the novel tank diving test (preference for greater depth). Zebrafish are also used in pharmacological studies to assess the effects of anxiolytics [22], stimulants [23], and alcohol [24], [25], [26], [27] as well as in studies of addiction [28], [18]. For example, chronic ethanol administration has been shown to disrupt shoaling [29], a common zebrafish response that can serve as a measure of social behaviour [30]. Ethanol consumption also leads to an increase in stress and anxiety during withdrawal after 7–8 days of constant [23] or once-daily [31] exposure. However, there are few studies examining the effects of longer term (>8 days) schedules of exposure on zebrafish behaviour.

The aim of the present study was to compare the behaviour of zebrafish after a long period of exposure to daily moderate doses and weekly high doses of ethanol. The schedules used were designed to approximate common patterns of alcohol consumption in humans. Over a 21-day period, we administered a low dose of ethanol once per day to a *daily-moderate exposure group*, and a high dose once per week to a *weekly-binge exposure group*, with each group receiving the same total dose across the three week period. After gathering baseline data using a modified light-dark paradigm [32] for testing scototaxis with naive fish, we then tested the experimental groups for light or dark preference, basic locomotor activity, and immobility on the second and ninth day of withdrawal.

## Method

### Animals and Housing

Adult wild-type (short fin) zebrafish (*Danio rerio)* were obtained from Big Al’s Aquariums and Fish Supply (Edmonton, AB) (n = 76). All fish were at least 9 months old and were housed in an Aquatic Habitats (AHAB, Aquatic Ecosystems, Inc. Apopka, FL, USA) three shelf bench top system with controlled filtration and aeration, in clear 3 L polypropylene tanks, at a density of seven to eight fish per tank. The fish were randomly assigned to one of four groups: naive (n = 32), control (n = 15), chronic (n = 15), or binge (n = 15). One fish from the binge group died from unknown causes. Fish were maintained on a 12 h light/dark cycle, with lights on at 8 AM and off at 8 PM, and 10% daily water changes. Luminance was measured in the habitat tanks with the lights on (33 cd/m^3^) with a cal SPOT 401 photometer (Cooke Corp. CA, USA). Fish were fed dry brine shrimp (Omega One Freeze Dried Mysis Shrimp nutri–treat, OmegaSea Ltd., Germany) or fish flakes (New Life Spectrum Optimum Fresh H_2_O Flakes, New Life International Inc. FL, USA) once per day on alternating days. Habitat water was buffered with non-iodized salt, sodium bicarbonate, and acetic acid. Daily water quality measures included temperature (which was maintained in the range of 26–28°C), pH (maintained at 7.0–8.0) and dissolved oxygen (maintained at 5–10 mg/l**)**. Weekly water quality measures included nitrates, nitrites, alkalinity, hardness, and conductivity.

### Ethanol Administration Procedure

**Figure 1:**
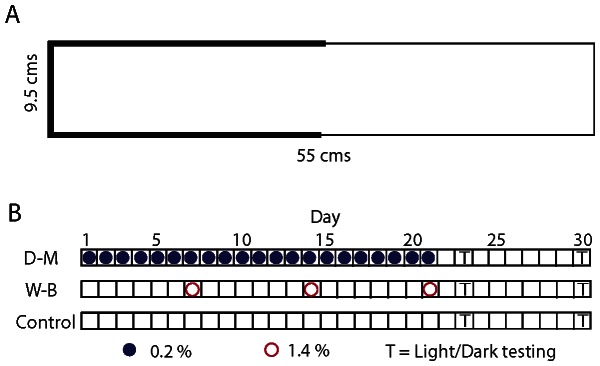
Scototaxis arena and ethanol administration schedule used with control, weekly-binge, and daily-moderate groups. (A) The scototaxis arena measured 9.5 cm wide, 9.5 cm deep, and 55 cm long, with black walls covering one half of the arena, white walls covering the other half, and white flooring throughout; water depth was maintained at 5 cm. (B) Ethanol administration and testing took place over a 30 day period. The daily-moderate group (•) received ethanol (0.2%) each day for 21 days of the exposure period, whereas the weekly-binge group (**○**) received ethanol (1.4%) only on the 7th, 14th and 21st days. All groups, including control, underwent the same daily procedures with the exception of ethanol administration. All animals were tested (T) on days 23 (2^nd^ day of withdrawal) and 30 (9^th^ day of withdrawal).

Prior to each dosing session, the fish in each group were netted and moved into a clear spawning tank containing a 400 micron insert (Aquatic Habitats) and normal habitat water. The inserts have small holes to allow zebrafish eggs to fall into the tank below. The inserts in the spawning tanks are also ideal for moving fish from normal water into another tank containing an ethanol solution. Lifting the spawning insert out of one tank and carefully submerging it in the dosing tank allows for rapid and simultaneous transfer of all fish in a group from one tank to another. This procedure ensured that all fish were exposed to ethanol for an identical duration (30 minutes). Ethanol was mixed with habitat water (26–28°C) in the dosing tanks at concentrations of 0% (control), 0.2% (daily-moderate), and 1.4% (weekly-binge) immediately before administration. Ethanol concentrations were based on previous studies [25], [31], [33]. Groups were split into two tanks of 7 or 8 fish per tank, with dosing being initiated across successive days for each group (Control Group 1, Weekly-Binge Group 1, Daily-Moderate Group 1, Weekly-Binge Group 2, Control Group 2, Daily-Moderate Group 2). This also allowed for scototaxis testing of each group to occur across successive days following the dosing period. The clear dosing tanks were placed on a table in a room with white walls, and the fish were monitored for signs of distress throughout the administration. All groups underwent the dosing procedure for 21 consecutive days, with the schedule of administration dependent on the group (Figure 1A). “Dosing” consisted of water only for 18 of the 21 days for the weekly-binge group and all 21 days for the control group. The daily-moderate and weekly-binge groups were exposed to the same total dose of alcohol by the end of the experiment. Ethanol administration occurred prior to daily feeding. Luminance in the dosing tank was 144 cd/m^3^.

### Scototaxis Testing Procedure

All experimental procedures were completed between 10 AM and 2 PM in a sound controlled room with diffuse overhead lighting. A three-sided enclosure of white corrugated plastic was constructed around the arena to minimize external visual stimuli. Fish were transferred in a habitat tank to the experimental room. After 10 minutes of acclimation, they were netted and placed in the center of the scototaxis arena. When released into the arena, the net was positioned parallel to the long axis of the arena to eliminate biasing the fish to either zone. The scototaxis arena was 9.5 cm wide by 55 cm long and 9.5 cm deep, and had a white plastic floor. The arena walls consisted of either white or black waterproof paper, which was affixed with Velcro to the sides of the tank (Figure 1B). We rotated the arena 180 degrees half-way through each day of testing to help eliminate possible bias as a result of differential visual stimuli due to arena orientation. Luminance was measured in the light zone (2.0 cd/m^3^), dark zone with the white floor (1.1 cd/m^3^), and dark zone with the black floor (0.0 cd/m^3^). Water in the testing arena was 5 cm deep and water temperature was maintained between 26–28°C. All trials were five minutes in duration. Data from fish that jumped out of the arena were discarded from the experiment (n = 4) and fish were not retested.

### Quantification of Behaviour

Zebrafish movement was tracked using the differencing method in Ethovision XT motion tracking software (version 7.0, Noldus, VA, USA). Quantification of behaviour began immediately after fish were placed in the center of the scototaxis arena. Elimination criteria included any trials with greater than 1.0% of the sample not found (no recordings met this criterion) or the fish jumping from the arena (control group at 2 days withdrawal, n = 2; weekly-binge group at 9 days withdrawal, n = 2). Time spent in zones (light or dark), number of zone transitions, and average velocity and immobility (5% threshold, based on Pham et al. [34]) were recorded. Data were analyzed using Graphpad Prism 4.0 B (CA, USA). All data was analyzed for normality with the D'Agostino & Pearson omnibus normality test. Since all data passed the normality test (alpha = 0.05) paired *t*-tests were used to test preference for light vs. dark. A preference index for light was also calculated by subtracting time spent in the light zone from time spent in the dark zone, with one sample *t*-tests used to assess statistically significant differences. Unpaired *t*-tests and one-way analysis of variance utilizing Tukey’s multiple comparison test were used to assess significant differences across groups. An alpha level of *p*<0.05 and 95% confidence intervals were used for assessing statistical significance in all tests.

### Ethics Statement

All experiments were approved by the Grant MacEwan University Animal Research Ethics Board (AREB) under protocol number 06-11-12, in compliance with the Canadian Council for Animal Care (CCAC) guidelines for the care and use of experimental animals.

## Results

### Baseline Scototaxis Preference in Naive Fish

**Figure 2:**
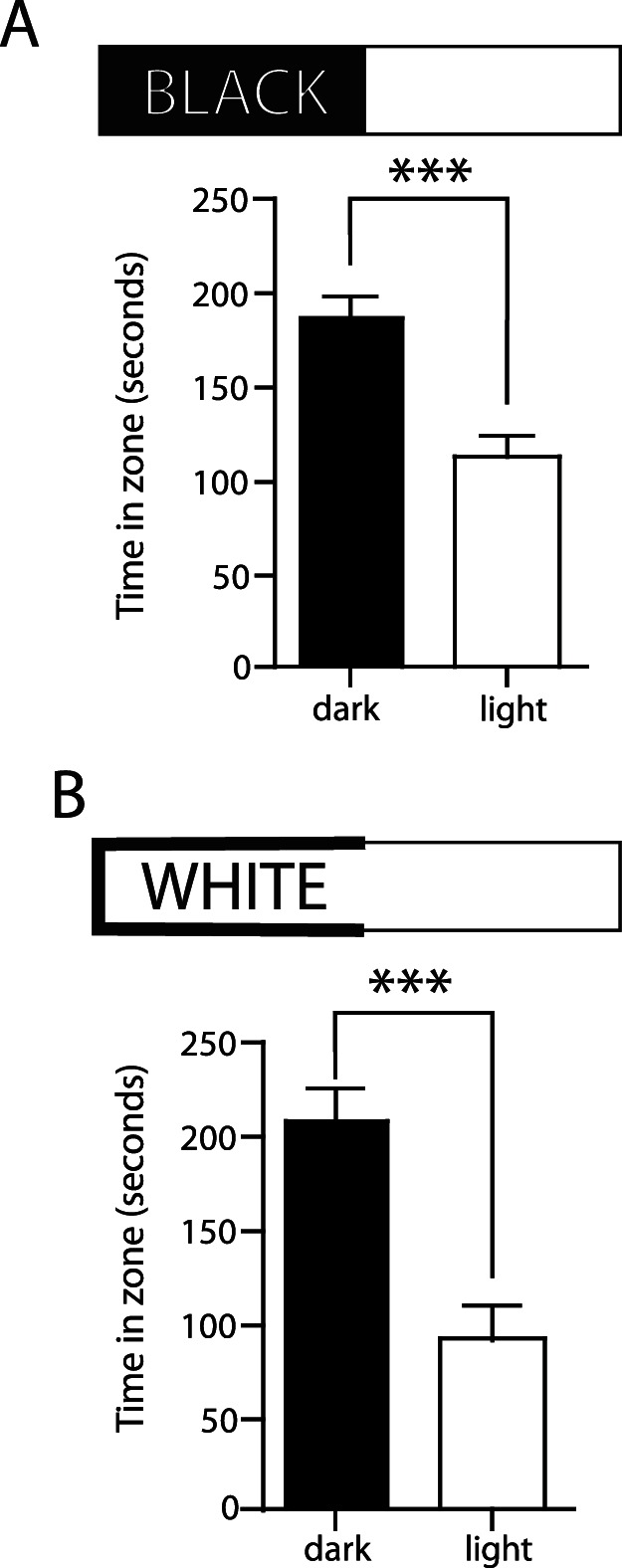
Naive preference for light/dark zones with and without a black floor. (A) When a black floor was used, zebrafish spent more time in the dark zone (186.9±11.16 sec, n = 20) compared to the light zone (113.1±11.16 sec, n = 20). (B) With a white floor, the fish also spent more time in the dark zone (207.8±15.37 sec, n = 12) compared to the light zone (92.22±15.37 sec, n = 12). ***p<0.001, paired *t*-test.

The scototaxis assay is a popular measure of anxiety in zebrafish. Previous studies have shown mixed results for naive fish, with juvenile fish strongly preferring light areas [35] and adult fish preferring dark areas [25], [31], [36]. The scototaxis paradigm typically involves a rectangular arena split into one dark half and one light half. However, the type of stimuli used to cover the floor of the arena differs across studies. Researchers have found a dark preference using black [32], [37] and sand covered floors [37] in the scototaxis assay, and clear floors in the light-dark plus maze [38]; conversely, a light preference has been observed with both transparent and opaque floors in the scototaxis assay [39], [40]. Therefore, we began by exploring whether the presence of a black versus white floor in the dark zone would have an impact on scototaxis in adult zebrafish. We first tested experimentally naive fish with the dark zone having both black sides and a black floor (Figure 2), and found that fish spent a significantly greater amount of time in the dark zone than the light zone (Figure 2A; dark zone: 186.9±11.16 s, n = 20; light zone: 113.1±11.16 s, n = 20; *p*<0.0001) as previously described. We then tested a second group of naïve fish with a dark zone that had a white floor and black sides. These fish also spent significantly more time in the dark zone (Figure 2B; dark zone: 207.8±15.37 s, n = 12; light zone: 92.22±15.37 s, n = 12; *p*<0.0001). There was no significant difference in dark zone preference between fish tested in the absence or presence of the black floor (*p* = 0.2730, unpaired *t*-test). Since motion-tracking is possible in the dark zone when white flooring is present in the entire arena, we used the dark zone with the black walls-white floor configuration for the remainder of the experiment.

### Scototaxis - Day Two of Withdrawal

**Figure 3:**
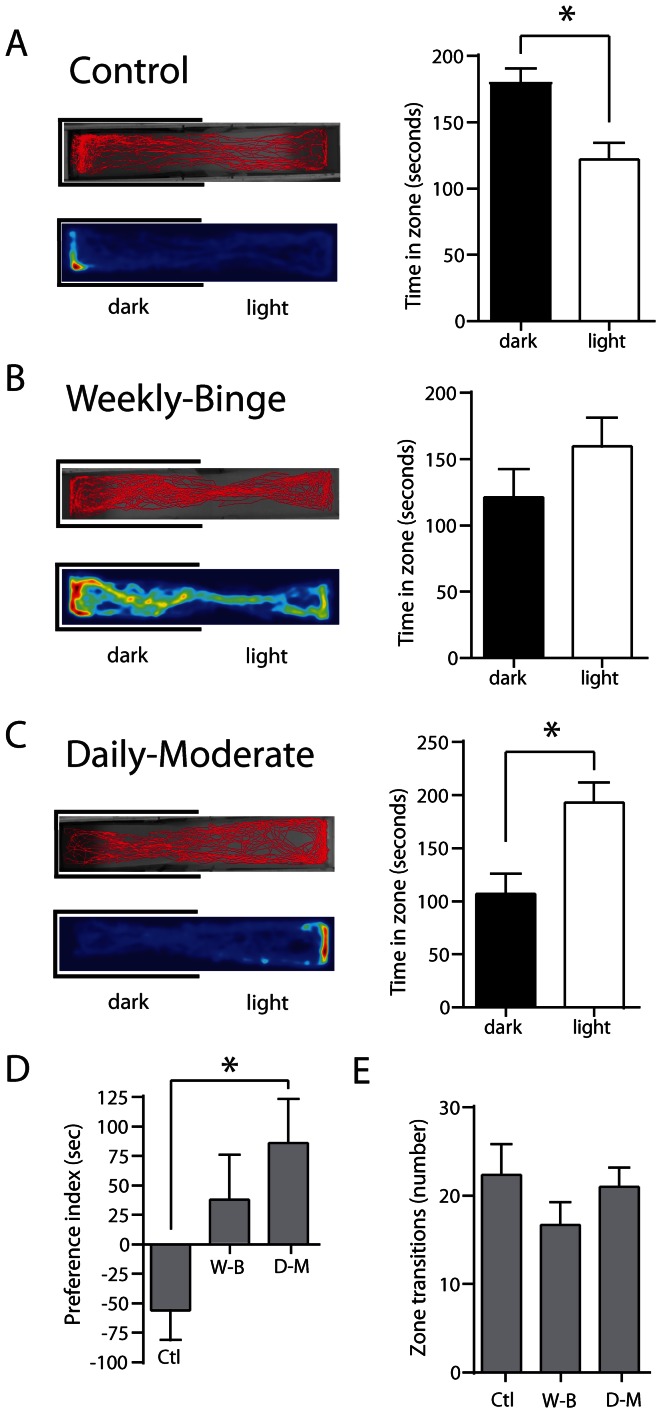
Daily-moderate ethanol exposure reverses scototactic preference on day two of withdrawal. (A) i: A plot of a single control zebrafish as it moved throughout the arena during the five minute trial. ii: Heatmap (pseudocolour representation of zebrafish movement) of the same fish throughout the five minute trial. iii: Total time spent in the light and dark zones for the five minute trial. Control fish preferred the dark zone (paired *t*-test, *p = *0.0442, n = 13). (B) i–ii: Same as in (A) but for a representative fish in the weekly-binge group. iii: Weekly-binge fish did not have a significant preference for either zone (paired *t*-test, *p = *0.3325, n = 14). (C) i–ii: Same as in (A) but for a representative fish in the daily-moderate group. iii: Daily-moderate fish spent significantly more time in the light zone (paired *t*-test, *p = *0.0366, n = 15). (D) Preference index for all groups. There was a significant difference in preference between daily-moderate and control groups (One way ANOVA, F (2,39) = 4.32, *p = *0.0202 with post-hoc Tukey test). *p<0.05. (E) The number of transitions between the dark and light zones for each group for the duration of the 5 minute trials. No significant differences in zone transitions were observed across all groups (One way ANOVA, F (2, 34) = 1.266, *p* = 0.2949).

To determine whether there was an effect of the schedule of ethanol administration on withdrawal-induced scototaxis, we tested the fish 2 days following the final dosing session with the scototaxis assay. Consistent with the naive fish in our baseline test, control fish which had not been exposed to ethanol spent significantly more time in the dark side of the arena (Figure 3A; dark: 178.1±12.5 s; light: 121.9±12.5 s; *p = *0.0442, n = 13). This effect was stronger for the naive fish than it was for the control group, (preference index - Naive: −115.6±30.7, n = 12, *p = *0.0032; Control: −56.17±25.0, n = 13, *p = *0.0442), although there was no significant difference between groups. The weekly-binge group showed no significant difference in time spent in the dark versus light zone (Figure 3B; dark: 121.4±20.91 s; light: 159.6±21.5 s, *p = *0.3325, n = 14), whereas fish exposed to a daily-moderate schedule spent significantly more time in the light zone (Figure 3C; dark: 107.0±18.6 s; light: 193.0±18.6 s, *p* = 0.0366, n = 15). We also found a significant difference in the preference index across the three experimental groups (one-way ANOVA, F (2, 39) = 4.32, *p = *0.0202; Figure 3D). A post-hoc Tukey’s multiple comparison test revealed a significant difference between control and daily-moderate groups (*p*<0.05), but no differences between control and weekly-binge groups (*p*>0.05) and daily-moderate and weekly-binge groups (*p*>0.05). There were no significant differences in the number of transitions from the light zone to the dark zone across all groups (F (2, 34) = 1.266, *p* = 0.2949; Figure 3E). There were no differences in average swimming velocity and immobility and no differences across groups (Table 1) indicating that ethanol withdrawal did not have a direct impact on basic locomotion.

**Table 1:** Average velocity and immobility during the scototaxis assay.

	Velocity(cm/s)		Immobility(sec)	
	2-days Wd	9-days Wd	2-days Wd	9-days Wd
Control (n = 13, 15)	9.1±0.6	9.4±0.6	1.9±0.6	2.2±0.7
Weekly-binge(n = 14, 12)	9.8±0.5	8.7±0.4	0.8±0.2	3.5±2.0
Daily-moderate(n = 15)	10.3±0.5	10.2±0.5	2.5±1.0	2.0±0.5

The average swim velocity (cm/s) and immobility (sec) was quantified with Ethovision XT for all groups during the scototaxis assay at 2 days and 9 days of withdrawal (Wd) from ethanol. There were no significant differences between or within all groups analyzed by one-way ANOVA. Velocity 2 days Wd: F (2, 39) = 1.288, *p* = 0.2873. Velocity 9 days Wd: F (2, 39) = 2.049, *p* = 0.1425. Immobility 2 days Wd: F (2, 38) = 1.457, *p* = 0.2456. Immobility 9 days Wd: F (2, 38) = 0.4554, *p* = 0.6376.

### Scototaxis - Day Nine of Withdrawal

**Figure 4:**
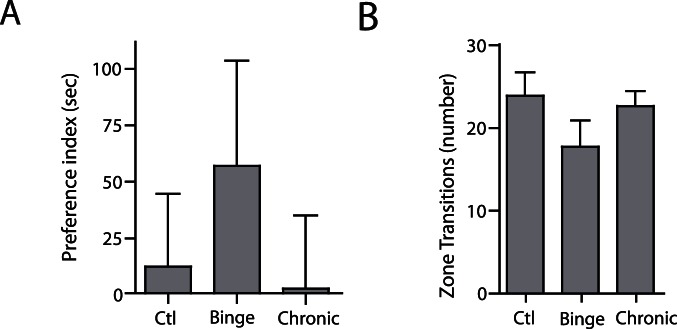
Dark preference and zone transitions after nine days of withdrawal. (A) The preference index was calculated as in Figure 3. No group showed a significant preference for light versus dark. There were no significant differences between groups (One way ANOVA, F (2, 39) *p* = 0.5487; n = 15 for control, n = 12 for weekly-binge, n = 15 for daily-moderate). (B) The number of zone transitions were calculated as in Figure 3. There were no significant differences in number of zone transitions between groups nine days after ethanol exposure. (One way ANOVA, F (2,39) = 1.592, *p = *0.2164; n = 15 for control, n = 12 for weekly-binge, n = 15 for daily-moderate).

To examine whether the group differences in scototaxis were transient or long-lasting, we repeated the scototaxis assay one week later, on day nine of ethanol withdrawal. We observed no significant differences in time spent in light versus dark zones for control (*p = *0.6979, n = 15), binge (*p = *0.2382, n = 12), or chronic (*p = *0.9301, n = 15) groups. Similarly, a one-way analysis of variance revealed no significant differences in preference index between the three groups (F (2, 39) = 0.6095 *p* = 0.5487; Figure 4A). Furthermore, there was no significant difference in the number of zone transitions across groups (one-way ANOVA, F (2, 39) = 1.592, *p* = 0.2164; Figure 4B), or in average swimming velocity or immobility (Table 1). Lastly, when we examined the shift in scototaxis preference from day 2 to day 9 in each group, we found no significant changes in preference index in the control (*p = *0.1065, n = 15) or weekly-binge (*p* = 0.3756, n = 12) groups; however, there was a significant decrease in light preference in the daily-moderate group between the two tests (*p = *0.0493, n = 15).

## Discussion

This study demonstrates that the schedule of ethanol dosing can have a significant impact on withdrawal-mediated behaviours. Previous research indicates that adult zebrafish prefer the dark area in the scototaxis assay [30], [31], [32], [41] which is consistent with the baseline results we obtained with naive fish. Regardless of whether the floor of the dark half of the arena was black or white, we consistently observed a naive preference for darkness. However, on day 2 of withdrawal, fish that were on a daily-moderate schedule of ethanol exposure spent significantly more time in the light zone compared to controls, whereas fish that were on a weekly-binge schedule showed no preference for dark or light. On day 9 of withdrawal, the daily-moderate group no longer showed a significant preference for the light zone. This indicates that there was a marked transient effect of daily low doses of ethanol relative to weekly high doses of ethanol on scototactic preference.

Our major finding is that withdrawal from a daily-moderate schedule of ethanol exposure can temporarily reverse the natural preference of zebrafish for the dark area in the scototaxis assay. A possible explanation for the day 2 withdrawal-induced preference for light is that ethanol is an appetitive stimulus for many animals, including zebrafish [24], [42], [43]. Additionally, rodents exposed to ethanol have been shown to search actively for ethanol and consume it freely if it is found [44], [45]. This may be due, in part, to an increase in glutamatergic neurotransmission that increases alcohol craving [3]. Thus, it is possible that zebrafish in the daily-moderate group exhibited a craving for alcohol during withdrawal that resulted in ethanol-seeking behaviour. This craving for ethanol could have subsequently facilitated the daily-moderate group’s preference for the light zone via processes of associative learning. In particular, the dosing procedure may have resulted in the development of a conditioned place preference (CPP). In a CPP procedure, contextual cues associated with an appetitive stimulus can increase an organism’s preference for that context. CPP has recently been shown in zebrafish, with the fish exhibiting preference for a compartment within which ethanol exposure had taken place [42], [46]. In the present study, the zebrafish were administered alcohol in transparent dosing tanks that were significantly more luminescent than their habitat tank. As a result, a relative increase in brightness might have become strongly associated with ethanol exposure. This might then have resulted in the daily-moderate group exhibiting a preference for the brighter side of the light-dark arena.

The daily-moderate exposure group demonstrated a light-zone preference at 2 days withdrawal, but not at 9 days withdrawal. This result is similar to what is found in rodent models, which show time dependent effects of ethanol withdrawal [48]. Furthermore, the daily-moderate group in the present study were exposed to the light-dark arena in the absence of ethanol during the day 2 withdrawal test, which would have constituted an extinction trial for any conditioned associations between relative brightness and ethanol administration. This stands in contrast to Kily et al. [47], who found enduring CPP effects in zebrafish during tests that took place 1 day, 7 days, and 22 days following their last ethanol exposure. The reason for this discrepancy may lie in both the greater number of conditioning trials that Kily et al. [47] used compared to the present study (28 days versus 21 days, respectively), as well as a higher dose of ethanol (0.5% versus 0.2%, respectively). Kily et al. [47] also tested for CPP using the same apparatus within which conditioning had taken place. In the present study, the light zone in the arena was only indirectly associated with the dosing chamber by virtue of being relatively brighter than the dark zone; this would have resulted in a relatively weak CPP that would more rapidly extinguish [49]. Based on our findings, it is reasonable to conceptualize the behavioural shift as resulting from a complex interaction of factors related to ethanol exposure (such as dose and pattern of administration) and factors related to extinction of conditioned preferences.

Dissimilar results can also be found in other studies of ethanol exposure. Methodological differences may again account for these discrepancies. Cachat and colleagues [23] reported anxiogenic effects of ethanol withdrawal in the Novel Tank Diving test; however, they used a 7-day constant exposure schedule, whereas we employed a 21-day, once-per-day exposure schedule. Mathur and Guo [31] used a once-per-day dosing procedure similar to ours, and found a dark preference after 7 days, but not at 1 day, of withdrawal. However, their fish were exposed to a considerably stronger ethanol solution (1% in their study versus 0.2% in our daily-moderate group) as well as for a shorter duration (8-days versus 21-days). These factors may have interfered with the development of an ethanol craving or a CPP.

Although there was no significant difference in preference between the weekly-binge group and the control group, the absence of the expected dark zone preference by the weekly-binge group is not readily explained. One possible explanation is that it reflects some degree of neurological impairment resulting from heavy ethanol exposure. Evidence indicates that cortical damage resulting from weekly-binge drinking can lead to a loss of inhibitory control and an increase in general impulsiveness and risk-taking [50]. The present results might be reflective of the same effect in zebrafish.

The control group showed the expected preference for the dark zone on day 2, but not day 9, of withdrawal. This suggests that the control group fish were further habituating to the light zone during repeated exposures to the arena. This can be contrasted with Maximino et al.’s (2010) findings of a lack of light zone habituation after 5 consecutive days of exposure to a light-dark arena. Methodological differences in the present study may account for this variation. The first is that the control group in the present study had already experienced 21 consecutive days of being transferred from the habitat to the very bright control dosing tank (144 cd/m^3^) for the 30 minute sessions. The naive fish were only exposed to the relatively dim habitat environment (33 cd/m^3^) and only handled during the scototaxis tests. This likely resulted in the control group having less of a preference than our naive group.

With respect to the scototaxis procedure employed in this study, our findings indicate that the color of the bottom of the arena did not affect scototactic behaviour during initial exposure to the arena. We found no significant difference in preference for the dark half of the arena when the floor was black versus white (Figure 1), suggesting that zebrafish respond mostly to the relative darkness of a zone as opposed to absolute darkness. This finding is advantageous for researchers using motion tracking software to quantify fish behaviour, since a dark bottom typically interferes with tracking. Parametric studies, however, are needed to determine the extent to which, as our results seem to suggest, differences in floor colouring may affect habituation in subsequent sessions.

In the present study, the fish were tested in the scototaxis assay 2 and 9 days following their final exposure to ethanol. This methodology differs from studies in which animals are tested immediately after or during an ethanol dosing procedure, and which typically result in an increase in activity [31], [36], [38], [39]. We found no differences during scototaxis testing in total distance moved or in the number of zone transitions from the light to dark zone across all groups on day 2 of withdrawal. This is likely due to the delayed testing procedure in the present study, which reduced the likelihood of any residual pharmacodynamic effects of ethanol during testing. It can therefore be assumed that any differences between our groups are due to past ethanol exposure and not the acute effects of ethanol itself.

Our results indicate that frequent low doses of ethanol produce significant changes in scototaxis in zebrafish and that these changes are greater than those caused by infrequent large doses of alcohol. The fact that our dosing schedule somewhat approximates two possible human drinking patterns– that of the frequent casual drinker and the infrequent binge drinker–suggests that the former schedule of consumption may be more likely to progress to alcohol addiction. These findings are consistent with the lack of alcohol-induced preference and withdrawal that is typically observed in human binge drinkers [3]. However, the findings also suggest that binge drinking may have adverse effects on risk-taking behaviour and impulsiveness. Finally, the present study confirms the use of the scototaxis assay with zebrafish as an effective paradigm for investigating both anxiety and the harmful effects of alcohol exposure.
